# Fast and Accurate Semi-Automated Segmentation Method of Spinal Cord MR Images at 3T Applied to the Construction of a Cervical Spinal Cord Template

**DOI:** 10.1371/journal.pone.0122224

**Published:** 2015-03-27

**Authors:** Mohamed-Mounir El Mendili, Raphaël Chen, Brice Tiret, Noémie Villard, Stéphanie Trunet, Mélanie Pélégrini-Issac, Stéphane Lehéricy, Pierre-François Pradat, Habib Benali

**Affiliations:** 1 Sorbonne Universités, UPMC Univ Paris 06, CNRS, INSERM, Laboratoire d’Imagerie Biomédicale, F-75013, Paris, Île de France, France; 2 APHP, Groupe Hospitalier Pitié-Salpêtrière, Service de Neuroradiologie, F-75013, Paris, Île de France, France; 3 Sorbonne Universités, UPMC Univ Paris 06, UMR-S975, Inserm U975, CNRS UMR7225, Centre de recherche de l’Institut du Cerveau et de la Moelle épinière—CRICM, Centre de Neuroimagerie de Recherche—CENIR, F-75013, Paris, Île de France, France; 4 APHP, Groupe Hospitalier Pitié-Salpêtrière, Département des Maladies du Système Nerveux, F-75013, Paris, Île de France, France; Centre Hospitalier Universitaire Vaudois Lausanne - CHUV, UNIL, SWITZERLAND

## Abstract

**Objective:**

To design a fast and accurate semi-automated segmentation method for spinal cord 3T MR images and to construct a template of the cervical spinal cord.

**Materials and Methods:**

A semi-automated double threshold-based method (DTbM) was proposed enabling both cross-sectional and volumetric measures from 3D T_2_-weighted turbo spin echo MR scans of the spinal cord at 3T. Eighty-two healthy subjects, 10 patients with amyotrophic lateral sclerosis, 10 with spinal muscular atrophy and 10 with spinal cord injuries were studied. DTbM was compared with active surface method (ASM), threshold-based method (TbM) and manual outlining (ground truth). Accuracy of segmentations was scored visually by a radiologist in cervical and thoracic cord regions. Accuracy was also quantified at the cervical and thoracic levels as well as at C2 vertebral level. To construct a cervical template from healthy subjects’ images (n=59), a standardization pipeline was designed leading to well-centered straight spinal cord images and accurate probability tissue map.

**Results:**

Visual scoring showed better performance for DTbM than for ASM. Mean Dice similarity coefficient (DSC) was 95.71% for DTbM and 90.78% for ASM at the cervical level and 94.27% for DTbM and 89.93% for ASM at the thoracic level. Finally, at C2 vertebral level, mean DSC was 97.98% for DTbM compared with 98.02% for TbM and 96.76% for ASM. DTbM showed similar accuracy compared with TbM, but with the advantage of limited manual interaction.

**Conclusion:**

A semi-automated segmentation method with limited manual intervention was introduced and validated on 3T images, enabling the construction of a cervical spinal cord template.

## Introduction

Spinal cord atrophy is a neuroimaging feature that is associated with motor neuron diseases such as amyotrophic lateral sclerosis (ALS) and spinal muscular atrophy (SMA) [1–5], inflammatory diseases such as multiple sclerosis (MS) [6–8], and spinal cord injuries (SCI) [9–11].

In the past two decades, several segmentation methods with varying degree of manual intervention have been proposed to assess cord atrophy from MR images [12–19]. These methods have been validated using mostly 1.5T images and mainly investigated cord atrophy in MS patients, at the upper cervical level where the spinal cord is most atrophied [20]. In the context of spinal cord diseases, there is clear interest in investigating larger portions of the spinal cord. Indeed, degenerative diseases of the spinal cord, such as ALS and SMA, are characterized by diffuse degeneration of lower motor neurons localized in the anterior horn of all spinal cord levels [21, 22]. The entire corticospinal tract also degenerates in ALS [22]. Spinal cord injuries can also affect all spinal cord levels [23].

In clinical practice, it is often difficult to visually determine spinal cord atrophy. The growing interest in assessing spinal cord atrophy using MRI therefore underlines the need for a fast and accurate method of spinal cord segmentation with limited manual intervention. Such method would indeed facilitate the investigation of large regions of the spinal cord using either volume or cross-sectional area (CSA) measurements. This method would also enable segmentation of large data sets in limited time. Furthermore, this would open the way to the construction of a template that could be used for cord normalization in group analyses. Several methods have been proposed to construct a template of the cervical spinal cord [24–26]. In [24, 26], less than 20 subjects were used to construct a T2-weighted 3T MRI template and such small sample was not representative enough of the anatomical variability of the spinal cord. In [24], a large amount of manual intervention was needed to straighten the spinal cord (one had to draw manually a line along the anterior edge of the spinal cord for each subject), while we introduce in this paper a segmentation method with minimal manual intervention. The cord straightening approach proposed in [24] leads to a better alignment of the anterior cord regions than the posterior cord regions, which is not the case in the present study as we use the cord centerline to straighten the spinal cord. Recently, a T1-weighted 1.5T MRI template has been proposed for the cervical spinal cord to quantify age-related atrophy as well as disease-related atrophy in MS patients [25, 27, 28]. However the authors used low-resolution 1.5T images and a semi-automated method, namely the active surface method (ASM) [14], which requires important manual intervention and lacks accuracy (CSA was reported to be overestimated by approximately 14% at C2 vertebral level compared with manual outlining).

In previous studies, the semi-automated threshold-based method (TbM) proposed in [18] proved highly accurate and reproducible at 1.5T (i.e., inter-, intra-operator and scan-rescan reproducibility) [14, 18, 29]. Recently, TbM has been validated at 3T in a large dataset of 111 subjects including healthy volunteers, ALS, SMA and SCI patients [30]. TbM underestimated CSA by 3.29% at the cervical level and 1.70% at the thoracic level. Interestingly, TbM proved highly robust to initialization suggesting potential automation.

The present study therefore has two main objectives: 1) To construct and validate a new fast and accurate segmentation method with minimal manual intervention benefitting from the high robustness of TbM to its initialization procedure; 2) As an application, to extend existing work at 1.5 and 3T [25, 26] to the construction of an accurate MRI template of the cervical spinal cord using a large sample of 3T images.

## Materials and Methods

### MRI acquisition

Subjects were positioned head-first supine, with a 2-centimeter-thick pillow to lift the head and no pillow below the neck. This strategy was used to limit the natural cervical cord lordosis at around C3–C4, i.e., excessive cord curvature in the antero-posterior direction. Subjects were positioned as comfortably as possible, centered in the scanner’s reference, and were systematically asked not to move during the acquisition in order to minimize motion artifacts.

Scans were performed at CENIR, Pitié-Salpétrière Hospital, Paris, France, using a 3T MRI system (TIM Trio 32-channel, Siemens Healthcare, Erlangen, Germany). The spinal cord was imaged using a three-dimensional T2-weighted turbo spin echo (TSE) sequence with a slab-selective excitation pulse (SPACE sequence: Sampling Perfection with Application optimized Contrasts using different flip angle Evolution). Imaging parameters were: voxel size = 0.9×0.9×0.9 mm^3^; Field of View (FOV) = 280×280mm²; 52 sagittal slices; Repetition time (TR)/ Echo time (TE) = 1500/120ms; flip angle = 140°; generalized autocalibrating partially parallel acquisition (GRAPPA) with acceleration factor R = 3; turbo factor = 69; acquisition time ~6 min. This sequence provides high signal-to-noise ratio due to 3D acquisition, high resolution due to isotropic acquisition, short acquisition times by combining parallel acquisition with high turbo factors. No specific absorption rate limitation was observed in any of our acquisitions. In 1.5T spinal cord MRI studies, SPACE sequence showed an absence of signal loss due to pulsatile CSF motion and no artifacts due to swallowing or vessel pulsation [31], improving clinical diagnosis potential of MRI [32] and an excellent assessment of anatomy and resistance to artifacts in imaging difficult anatomy such as in scoliosis and spondylolysis [33], compared with conventional MRI sequences. At 3T, SPACE sequence showed an increased signal to noise ratio compared with 1.5T [34, 35] and allowed better inter-observer agreement for detecting foraminal stenosis, central spinal stenosis, and nerve compression compared with 2D T2-weighted TSE sequence [36].

### Segmentation method

#### Preprocessing

Data were corrected for B_1_ non-uniformity intensity using Minc-Toolkit N3 [37]. Preprocessing of MR images using N3 has been shown to substantially improve the accuracy of anatomical analysis techniques such as tissue classification and segmentation as well as cortical surface extraction [38, 39]. The operator had to choose a region of interest interactively using an in-house Matlab script (The Mathworks Inc., Natick, MA, USA), as follows: The operator just had to display the sagittal slices where the spinal cord was the most median in the FOV, and to select with two mouse clicks the upper and lower limits of the spinal cord region to be segmented; the images were then automatically cropped to focus on this region of interest. This was the only manual intervention required in the whole segmentation procedure. The produced images were then resampled to a voxel size of 0.3×0.3×0.3 mm^3^ using 3D cubic interpolation in order to maximize segmentation accuracy [11, 30].

#### Segmentation: Volumetric approach

**Fig 1:**
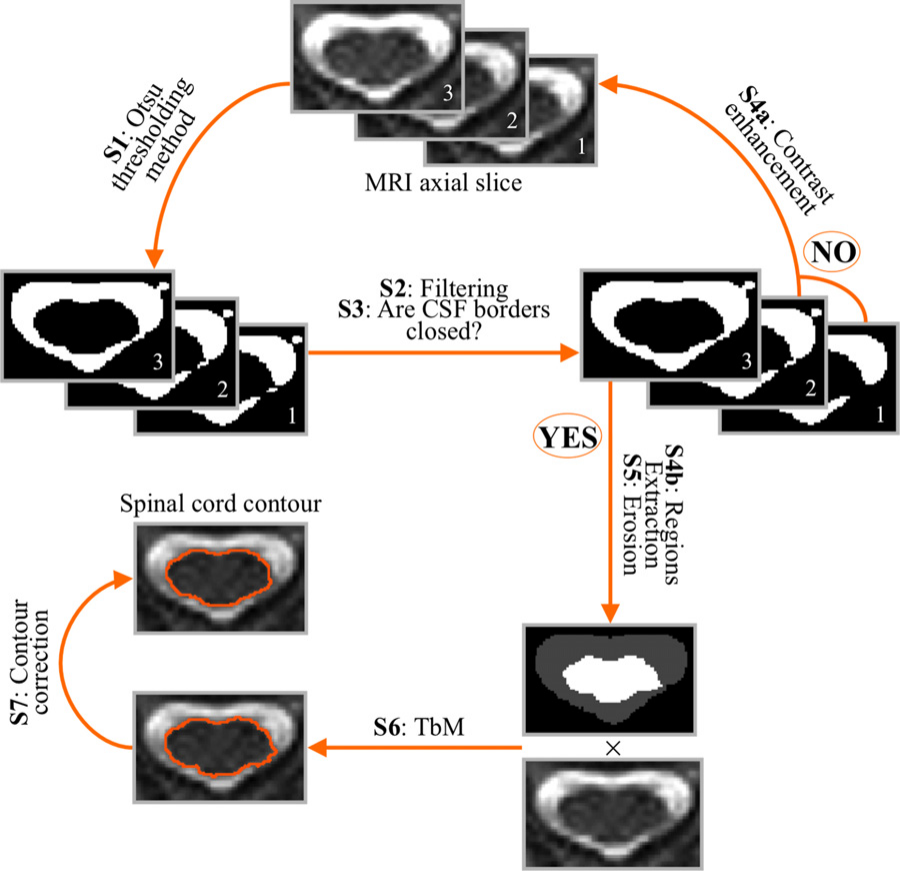
Overview of DTbM steps (S). Slice 1 represents an example of a resampled axial image. Slices 2 and 3 represent the progressive contrast enhancement of slice 1.

Conventional TbM [18] requires manual initialization since the operator has to delineate in each axial slice to be segmented the contours of the CSF and spinal cord regions. TbM then uses the mid intensity of the mean intensities in these regions as a threshold to classify voxels into two classes, namely spinal cord and CSF. By conducting numerical simulations, we have shown previously that TbM was highly robust to its initial contours [30]. More specifically, simulations suggested optimal segmentation results would be obtained with initial contours representing as little as 10% to 30% of manually drawn areas for both spinal cord and CSF. Full automation of the TbM method could therefore be envisaged by automatically extracting CSF and cord regions, while allowing 10% to 30% error rate as compared with manually drawn areas. This led us to design a double threshold-based method with minimal manual intervention (DTbM) implemented using Matlab, which involved the following steps in each slice of the region of interest to be segmented (see Fig. 1 for a flowchart of the method):

S1: The slice was segmented using Otsu’s global thresholding method (first threshold of DTbM) [40]. This method seeks for the optimal grey level threshold that maximizes the inter-class variance between the foreground and the background of the MRI images. The optimal threshold enables to select pixels of highest intensity, corresponding mainly to CSF.

S2: A binary mask M_CSF_ of the CSF was obtained by selecting from these pixels the cluster with the highest intensity and a minimum size of 50 connected pixels.

S3: A flood-fill algorithm [41] was applied in order to fill the inner part of M_CSF_, yielding to a mask M_F_.

S4a: If M_F_-M_CSF_ was zero, this meant that the border of the CSF mask was not completely closed and there was no inner region to fill. The global contrast of the initial MRI slice was then enhanced by truncating the image histogram by 0.1% of its upper end and then steps S1 to S3 were repeated until M_F_-M_CSF_ was not zero, which meant that the border of the CSF mask was closed and the inner part had been completely filled.

S4b: If M_F_-M_CSF_ was not zero, this difference between M_F_ and M_CSF_ corresponded to the inner part of the CSF mask, i.e. a binary mask of the spinal cord (M_SC_).

S5: M_SC_ and M_CSF_ were eroded (disk radius of 3 pixels) in order to lie between 10% to 30% error rate as compared with the expected manually drawn areas [30].

S6: CSF and spinal cord regions obtained by masking the initial image by the binary masks from S5 were taken as initial inputs to the threshold-based method (TbM) proposed by Losseff et al. [18], as demonstrated in [30]. TbM uses the mid intensity of the mean intensities in the two regions as a threshold (second threshold of DTbM) to further classify region pixels into two classes, namely the spinal cord and the CSF [18]. CSA was defined as the mean area in mm^2^ of the resulted masks.

S7: The segmented spinal cord regions obtained from S6 in all slices were corrected in order to adjust for possible missing or inaccurate segmentations. First, the centerline of the spinal cord was extracted by locating the center of mass of the spinal cord region in each slice. The centerline curve was smoothed using robust locally weighted regression [42]. The derivative of the centerline and polynomial curve fitting were used to detect aberrant discontinuities in the centerline then to remove them (a threshold of 15 voxels was set). Missing points in the centerline were then estimated using cubic spline interpolation. The radius from the center of mass of each axial mask to its borders was measured with an angular resolution of 5° by mapping Cartesian coordinates to polar coordinates (radius every 5°) [11]. To limit nerve roots contribution to the final segmentation, the length of the radiuses was smoothed for each angle using robust locally weighted regression across slices. Then, radiuses of the missing masks were estimated for each angle using cubic spline interpolation across slices. Finally, the contours of the spinal cord were calculated by converting polar coordinates (center of mass and radius every 5°) back to Cartesian coordinates. Finally, contours were converted to binary masks leading to a 3D mask covering the whole spinal cord region.

#### Segmentation: cross-sectional area approach

When only a few slices needed to be segmented, the volumetric approach was replaced by a cross-sectional area approach, which merely consisted of segmenting preprocessed images using steps S1 to S6 as described above.

### Comparison with existing methods

First, the volumetric measures obtained from DTbM in all spinal cord vertebral levels were compared with those obtained from ASM [14] using visual scoring. Second, the CSA measures obtained from DTbM at C2 vertebral level were compared with those obtained from ASM and TbM [18] using quantitative criteria, taking manual outlining as the ground truth. All segmentations were achieved by an experienced operator (i.e. four years’ experience) using in-house Matlab scripts for manual outlining, DTbM and TbM and a trial version of Jim 6.0 (Xinapse Systems, UK; http://www.xinapse.com) for ASM. ASM is based on image smoothing followed by an active surface model constrained by intensity gradient image information. The operator placed landmarks on the center of the spinal cord in the axial plane at the extremes of the cord region to be segmented and (i) every 10 axial slices for volumetric approach and (ii) in all axial slices for CSA measures. The parameters of the method were: nominal cord diameter: range = 7–10 mm; number of shape coefficients = 24; order of the longitudinal variation = 12. For parameter adjustment, the experienced operator was guided by the user’s manual of the method (http://www.xinapse.com/Manual/), data characteristics as well as visual assessment of segmentation results.

### Subjects

One hundred and twelve subjects were randomly selected from our database of spinal cord 3T MRI scans. There were 82 healthy volunteers, 10 patients with ALS, 10 patients with SMA and 10 patients with SCI. Four healthy subjects were excluded from our study due to disk protrusion compressing the spinal cord (n = 2, at C5/C6 level) or inducing loss of CSF space and contacting the spinal cord (n = 2, at C3/C4, C4/C5 and C5/C6 levels). The local Ethics Committee of our institution approved all experimental procedures (Paris-Ile de France Ethical Committee under the 2009-A00291-56 registration number), and written informed consent was obtained from each participant. MRI experiments were conducted according to the principles expressed in the latest revision of the Declaration of Helsinki.

### Evaluation

#### Groups

MR images from the healthy subjects comprised two fields of view, one covering the whole cervical and the upper thoracic spinal cord regions (n = 60) and the other including partly the cervical and the thoracic spinal cord regions (n = 18). MR images from the patients covered the whole cervical and upper thoracic spinal cord regions. Therefore, subjects were split into two groups:

- Group 1 included 7 subjects randomly chosen from the 60 healthy volunteers with images covering the whole cervical and the upper thoracic spinal cord, 3 subjects randomly chosen from the 18 healthy volunteers with images including partly the cervical and the thoracic spinal cord (mean age of selected healthy subjects ± SD: 41.9 ± 16.5 years, 4 females), 10 patients with ALS (mean age ± SD: 54.5 ± 10.9 years, 3 females), 10 patients with SMA (mean age ± SD: 36.1± 10.9 years, 8 females), and 10 patients with SCI (mean age ± SD: 46.4±18.2 years, 2 females). Table 1 shows the segmented spinal cord region for each subject. Their images were used for visual evaluation of DTbM and ASM segmentations using the volumetric approach at the cervical and thoracic spinal cord regions. They were also used for quantitative evaluation of CSA accuracy measured at the middle of each available spinal cord vertebral level (Table 1).
- Group 2 included 10 healthy volunteers randomly chosen from the 60 healthy subjects with images covering the whole cervical spinal cord (mean age ± SD: 45.7 ± 15.8 years, 6 females), and the same ALS, SMA and SCI patients as in Group 1. Their images were used for quantitative comparison of DTbM, TbM and ASM segmentations at C2 vertebral level. This region had high contrast between CSF and spinal cord as well as large CSF spaces and low CSA variability, and therefore segmentation was expected to be more reproducible and accurate at this level. Furthermore, recent studies measuring CSA have shown pronounced cord atrophy at C2 vertebral level in ALS and SCI patients that correlates with clinical disability [1, 10, 11].

**Table 1:** Segmented spinal cord regions for each group of subjects (Upper–Lower vertebral level limits).

Controls	ALS	SMA	SCI
C2–T5	C2–T2	C2–T5	C2–T4
C7–T9	C2–T3	C2–T4	C2–T3
C2–T4	C2–T5	C2–T6	C2–C7
C3–T8	C2–T4	C2–T5	C2–T3
C2–T1	C2–T1	C2–T6	C2–T1
C3–T8	C2–T5	C2–T6	C2–T3
C2–T4	C2–T5	C2–T5	C2–T4
C2–T3	C2–T5	C2–T5	C2–T2
C2–T4	C2–T6	C2–T4	C2–T4
C2–T6	C2–T4	C2–T5	C2–T4

All data were preprocessed using the method described in the “Preprocessing” section above.

#### Visual evaluation (volumetric approach)

Images from Group 1 were segmented using the volumetric version of DTbM (steps S1 to S7) and ASM [14]. Accuracy of DTbM and ASM segmentations was evaluated visually by an independent expert neuroradiologist (with seven years’ experience) using the OsiriX Imaging Software (http://www.osirix-viewer.com/). The operator was blinded to the type of data and methods. The evaluation used a combined score based on neuroradiological diagnosis and overlap criteria (Table 2): The operator evaluated visually the overlap between the spinal cord region and the mask resulting from each segmentation method in the axial plane, in the whole spinal cord in the FOV (Table 1, global overlap) and in specific regions of the spinal cord that were subject to T_2_-hyperintensity, atrophy, or narrow spinal canal, which are usually present in clinical routine MR images of the pathological spinal cord. The operator also evaluated segmentation accuracy in visually normal appearing spinal cord regions and nerve roots regions. The overlap criteria were:

- Insufficient: The method failed to segment accurately the spinal cord. The resulted axial masks underestimated/overestimated by a large number of voxels the visually expected masks.
- Moderate: The resulted axial masks were close to but partially larger or smaller than the expected spinal cord region.
- Substantial: The resulted axial masks underestimated/overestimated the visually expected spinal cord region by only few voxels.
- Perfect: Resulted axial masks fitted perfectly the spinal cord region.
- Included: Resulted axial masks included voxels from nerve roots.
- Not included: Resulted axial masks did not include voxels from nerve roots.

**Table 2:** Criteria and scores for the visual evaluation.

Criteria	Score
1- Global overlap agreement	
Insufficient to moderate	0
Substantial	1
Perfect	2
2- Overlap agreement by region	
Normal appearing spinal cord	
Insufficient^1^	0
Moderate^2^	1
Substantial^3^	2
Perfect^4^	3
Nerve roots	
Included	0
Not included	1
T2-hyperintensity	
Insufficient to moderate	0
Substantial to perfect	1
Atrophy	
Insufficient to moderate	0
Substantial to perfect	1
Narrow spinal canal	
Insufficient to moderate	0
Substantial to perfect	1

^1^Insufficient to moderate overlap in at least 3 vertebral levels.

^2^Insufficient to moderate overlap in less than 3 vertebral levels.

^3^Global substantial overlap agreement.

^4^Global perfect overlap agreement.

#### Quantitative evaluation

Quantitative evaluation of CSA measures obtained on the whole spinal cord using the volumetric approach would have required manual segmentation of a large number of slices to get a ground truth, which was untractable. Nevertheless, to try and evaluate the performance of the volumetric approach at all cord levels, a ground truth was obtained by manually segmenting the middle slice of each available spinal cord vertebral level (Table 1) for all subjects of Group 1. In the SCI group, only vertebral levels rostral and caudal to the spinal cord lesions were considered [10, 30]. CSA values obtained in this middle slice using DTbM (see section “Segmentation: volumetric approach” above) and ASM were compared with manual outlining.

On the other hand, we carried out a specific evaluation of the cross-sectional approach at C2 vertebral level using images from Group 2, in order to conform to segmentation evaluations largely conducted in the literature. This required straightening the spinal cord before segmentation, as follows: the cord centerline was extracted (i.e. the line linking the centers of mass of all axial masks) and further smoothed using robust locally weighted regression. Spinal cord images were resampled in planes perpendicular to the centerline using 3D cubic interpolation leading to straight spinal cord images. Five three-millimeter-thick slices were selected with the most inferior slice passing centrally through the C2/C3 disk, as largely made in MS and SCI studies at C2 vertebral level [7–10, 14, 18, 43–46]. Unlike what has been proposed in [16, 43], we chose to resample data to account for partial volume between the spinal cord and the CSF as proposed in [11, 30]. In addition, cord straightening enabled to reduce partial volume due to the cord orientation between the CSF and the spinal cord as well as blurring at the interface between the two tissues, and thus increase segmentation accuracy [16, 43]. Images were segmented using manual segmentation, TbM, DTbM (as described in “Cross-sectional area approach” section above), and ASM.

Accuracy was evaluated in two ways: (1) by using the Dice similarity coefficient (DSC), which evaluates the performance of segmentation methods by measuring their spatial overlaps [47]; the DSC is defined as follows: 2×|GT ∩ S| ⁄ (|GT| + |S|), where |GT| and |S| are the CSA in mm^2^ obtained by the manual segmentation (ground truth) and the segmentation method to be evaluated (DTbM, TbM or ASM), respectively. |GT ∩ S| is the area in mm^2^ common to both segmentation results; (2) by computing the relative CSA estimation error made by DTbM, TbM and ASM, considering the CSA from manual segmentation made by the experienced operator as the ground truth.

### Template construction

The 60 healthy subjects with images covering the whole cervical spinal cord (mean age ± SD: 44.3 ± 16.2 years, range: 21–72 years, 31 females) were used to construct the cervical spinal cord template.

#### Preprocessing

The Minc-Toolkit N3 tool [37] was used to correct MR images for intensity non-uniformity then to apply intensity normalization. The experienced operator defined manually two landmarks in the sagittal slice where the spinal cord was the most median in the FOV to delimit the cervical spinal cord (i.e. from the upper limit of C2 vertebral level to the middle vertebral body C7/T1). After cropping and resampling the images as described in the “Volumetric approach: preprocessing” section above, a second non-uniformity intensity correction was applied using N3 in order to remove residual artifacts.

#### Segmentation

The cervical spinal cord was segmented using the volumetric version of DTbM (steps S1 to S7) and the cord centerline was extracted (i.e. the line linking the centers of mass of all axial masks) and further smoothed using robust locally weighted regression.

#### Standardization

Spinal cord images and cord masks were resampled in planes perpendicular to the centerline using 3D cubic interpolation for cord images and 3D nearest neighbor interpolation for masks, the centerline being centered in the FOV, which avoided registration between individuals as done in [25]. Thereby, the spinal cord images were always centered in the antero-posterior and left-right directions. Resulting images were standardized in length by rescaling them to the median cord length using 3D cubic interpolation for cord images and 3D nearest neighbor interpolation for masks (median length across subjects = 402 slices) as proposed in [25].

#### Template and probability tissue map

Straightened cord images were averaged across subjects and smoothed using 3D Gaussian filter (FWHM = 0.3×0.3×0.6 mm) to insure the validity of the Gaussian random theory, as well as to correct for possible imperfections in the standardization process, as showed in [25], which yielded a template of the cervical spinal cord. Straightened cord masks were averaged, which yielded a tissue probability map for the spinal cord, where voxel value from 0 to 1 is the probability for the voxel to belong to the spinal cord.

## Results

Statistical analyses were conducted with Matlab software. Wilcoxon signed-rank test was used to compare accuracy measurement of DTbM, TbM and ASM at C2 vertebral level, and DTbM and ASM at the cervical and thoracic levels; p values less than 0.05 were considered significant.

### Computational time

**Fig 2:**
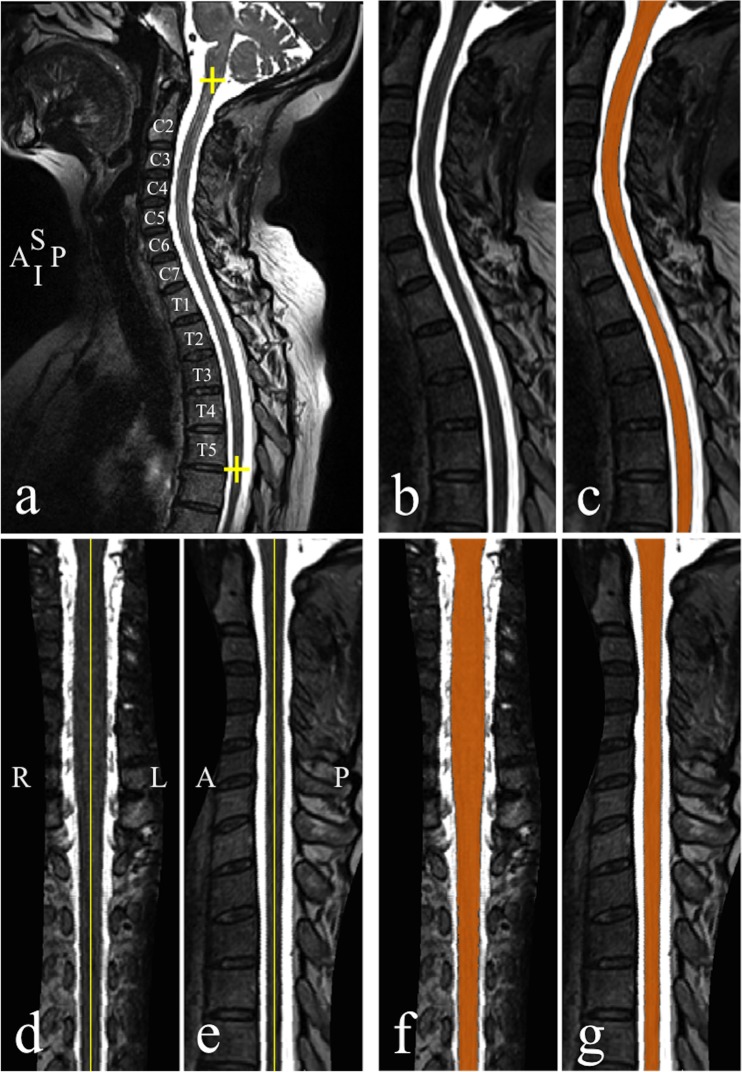
Segmentation and cord straightening. (a) T2-weighted mid-sagittal section in an ALS patient. The yellow plus signs delimit the spinal cord region to be preprocessed and segmented. (b) Mid-sagittal section of the preprocessed data and (c) resulted segmentation mask (orange). (d) Mid-coronal and (e) sagittal sections of the straightened spinal cord. (f) Mid-sagittal and (g) coronal sections of the straightened mask (orange). A, anterior; I, inferior; L, left, P, posterior, R, right, S, superior.

The mean number (± SD) of segmented slices was 701±96 slices/subject. Computation time for the whole procedure was 3.9±1.2 min/subject (mean ± SD), which included preprocessing time: 38±4 s/subject, segmentation time: 1.9±1.1 min/subject, correction (step S7) time: 1.5±0.3 min/subject. This was achieved using a 64-bit Quad-core (Intel Xeon, processor speed: 2.67 GHz) workstation. Computation time was not optimized as the whole procedure was coded in Matlab language. Fig. 2 shows an example of segmentation result from DTbM in an ALS patient.

### Visual evaluation (volumetric approach)

Accuracy results are shown in Table 3 for each group of subjects. DTbM outperformed ASM in both healthy and pathological groups for global overlap (number of scores for DTbM/ASM: 33/1 perfect, 7/31 substantial and 0/ 8 insufficient to moderate), overlap in normal appearing spinal cord (DTbM/ASM: 28/0 perfect, 11/8 substantial, 1/11 moderate and 0/21 insufficient) and nerve roots regions (DTbM/ASM: 39/36 not included and 1/4 included in the final segmentations). Both DTbM and ASM showed similar overlap performance in regions with T_2_-hyperintensity (DTbM/ASM: 6/4 substantial to perfect and 5/7 insufficient to moderate), atrophy (DTbM/ASM: 5/3 substantial to perfect and 4/6 insufficient to moderate) and narrow spinal canal (DTbM/ASM: 3/3 substantial to perfect and 3/3 insufficient to moderate). The individual visual scoring is shown in S1 Table.

**Table 3:** Results of the visual evaluation.

Criteria	Number of scores for DTbM/Number of scores for ASM			
	Controls	ALS	SMA	SCI
1- Global overlap agreement				
Insufficient to moderate	0/2	0/2	0/3	0/1
Substantial	2/8	1/8	1/7	3/8
Perfect	8/0	9/0	9/0	7/1
2- Overlap agreement by region				
Normal appearing spinal cord				
Insufficient	0/5	0/5	0/7	0/4
Moderate	0/4	0/3	0/2	1/2
Substantial	3/1	2/2	3/1	3/4
Perfect	7/0	8/0	7/0	6/0
Nerve roots				
Included	0/1	1/1	0/2	0/0
Not included	10/9	9/9	10/8	10/10
T2-hyperintensity				
Insufficient to moderate	0/1*	0/1	1/0	4/5
Substantial to perfect	1/0*	1/0	0/1	4/3
Atrophy				
Insufficient to moderate	-	0/2	0/0	4/4
Substantial to perfect	-	2/0	1/1	2/2
Narrow spinal canal				
Insufficient to moderate	-	0/1	1/1	2/1
Substantial to perfect	-	1/0	1/1	1/2

*Asymptomatic syrinx revealed by the visual evaluation.

### Quantitative evaluation

#### Volumetric approach

Table 4 shows accuracy measurements at the cervical and thoracic levels. At the cervical level, the mean DSC value was 95.71% for DTbM and 90.78 for ASM (manual outlining was considered as the ground truth). At the thoracic level, the mean DSC value was 94.27% for DTbM and 89.93% for ASM. When considering separately each group of subjects, the mean DSC value was 96.07% for DTbM and 90.89% for ASM in the control group. In the ALS group, the mean DSC value was 95.71% for DTbM and 90.06% for ASM. In the SMA group, the mean DSC value was 95.30% for DTbM, 91.07% for ASM. Finally, in the SCI group, the mean DSC value was 95.75% for DTbM and 91.10% for ASM.

**Table 4:** Accuracy of mean cross-sectional area measured at the cervical and thoracic levels.

	Cervical level		Thoracic level	
	Method		Method	
Accuracy measurements	DTbM	ASM	DTbM	ASM
Mean (SD) dice similarity coefficient (%)				
Whole population	95.71 (1.00)	90.78 (2.85)	94.27 (4.31)	89.93 (4.21)
Controls	96.07 (0.81)	90.89 (3.62)	95.33 (0.8)	89.57 (3.55)
ALS	95.71 (1.19)	90.06 (2.75)	94.91 (1.04)	90.74 (2.57)
SMA	95.30 (1.01)	91.07 (2.51)	95.12 (0.84)	91.03 (1.60)
SCI	95.75 (0.93)	91.10 (2.73)	91.07 (8.95)	88.00 (7.65)
Mean (SD) error estimation (%)				
Whole population	-4.86 (3.68)	13.53 (6.73)	-6.49 (3.42)	9.47 (7.87)
Controls	-5.05 (2.41)	14.34 (7.32)	-4.81 (3.00)	16.08 (9.49)
ALS	-4.38 (4.20)	15.44 (6.48)	-6.87 (2.37)	14.99 (6.92)
SMA	-4.39 (5.20)	13.88 (9.05)	-6.72 (2.62)	15.26 (4.28)
SCI	-5.61 (2.63)	11.08 (4.28)	-7.54 (4.96)	12.08 (8.47)

Manual segmentation by the experienced operator was the ground truth for accuracy measurements.

DTbM underestimated the mean CSA measured by manual segmentation (considered as the ground truth) by 4.86% and 6.49% and ASM overestimated the mean CSA measured by manual segmentation by 13.53% and 9.47% at the cervical and thoracic levels, respectively. When considering separately each group of subjects, DTbM underestimated the mean CSA measured by manual segmentation by 5.05% and 4.81% and ASM overestimated the mean CSA by 14.34% and 16.08% at the cervical and thoracic levels, respectively. In the ALS group, DTbM underestimated the mean CSA measured by manual segmentation by 4.38% and 6.87% and ASM overestimated the mean CSA by 15.44% and 14.99% at the cervical and thoracic levels, respectively. In the SMA group, DTbM underestimated the mean CSA measured by manual segmentation by 4.39% and 6.72% and ASM overestimated the mean CSA by 13.88% and 15.26% at the cervical and thoracic levels, respectively. Finally, in the SCI group, DTbM underestimated the mean CSA measured by manual segmentation by 5.61% and 7.54% and ASM overestimated the mean CSA by 11.08% and 12.08% at the cervical and thoracic levels, respectively. The behaviour of DTbM method is illustrated in S1 Fig.

Based on DSC values, DTbM showed a significantly higher accuracy for the whole population than ASM at both the cervical and thoracic levels (Wilcoxon signed-rank test, p<0.001).

#### Cross-sectional area approach at C2 vertebral level

Table 5 shows mean CSA measurements made by the experienced operator using manual outlining, as well as DTbM, TbM and ASM results. Results of accuracy are also given. The mean DSC value was high for DTbM (97.98%), TbM (98.02%) and ASM (96.76%). When considering separately each group of subjects, the mean DSC value was 98.18% for DTbM, 98.2% for TbM and 96.63% for ASM in the control group. In the ALS group the mean DSC value was 97.86% for DTbM, 97.92% for TbM and 96.83% for ASM. In the SMA group the mean DSC value was 98.01% for DTbM, 98.11% for TbM and 96.83% for ASM. Finally, in the SCI group, the mean DSC value was 97.86% for DTbM, 97.87% for TbM and 96.77% for ASM.

**Table 5:** Accuracy of mean cross-sectional area measured at C2 vertebral level.

	Method		Method	
Accuracy measurements	Manual	DTbM	TbM	ASM
Mean (SD) CSA (mm^2^)	81.07 (13.3)	80.97 (14.08)	80.86 (13.96)	85.19 (14.52)
Mean (SD) Dice similarity coefficient (%)				
Whole population	–	97.98 (0.43)	98.02 (0.38)	96.76 (0.85)
Controls	–	98.18 (0.33)	98.2 (0.31)	96.63 (1.14)
ALS	–	97.86 (0.53)	97.92 (0.44)	96.82 (0.98)
SMA	–	98.01 (0.45)	98.11 (0.39)	96.83 (0.64)
SCI	–	97.86 (0.34)	97.87 (0.34)	96.77 (0.65)
Mean (SD) error estimation (%)				
Whole population	–	-0.25 (2.13)	-0.36 (1.90)	5.02 (3.62)
Controls	–	0.34 (1.87)	0.10 (1.78)	6.21 (2.76)
ALS	–	0.25 (2.64)	0.11 (2.37)	5.07 (3.69)
SMA	–	-0.63 (2.08)	-0.74 (1.64)	4.66 (3.75)
SCI	–	-0.96 (1.88)	-0.91 (1.79)	4.13 (4.35)

Manual segmentation by the experienced operator was the ground truth for accuracy measurements.

DTbM and TbM underestimated the mean CSA measured by manual segmentation (considered as the ground truth) by 0.25% and 0.36%, respectively. ASM overestimated the mean CSA measured by manual segmentation by 5.02%. When considering separately each group of subjects, DTbM, TbM and ASM overestimated the mean CSA measured by manual segmentation by 0.34%, 0.10% and 6.21%, respectively, in the control group. In the ALS group, DTbM, TbM and ASM overestimated the mean CSA measured by manual segmentation by 0.34% for DTbM, 0.10% for TbM and 6.21% for ASM. In the ALS group, DTbM, TbM overestimated the mean CSA measured by manual segmentation by 0.25% for DTbM and 0.11% for TbM and 5.07%. In the SMA group, DTbM, TbM underestimated the mean CSA measured by manual segmentation by 0.63% for DTbM and 0.74% for TbM. ASM overestimated the mean CSA measured by manual segmentation by 4.66%. Finally, in the SCI group, DTbM, TbM underestimated the mean CSA measured by manual segmentation by 0.96% for DTbM and 0.91% for TbM. ASM overestimated the mean CSA measured by manual segmentation by 4.13%.

Based on DSC values, DTbM showed a significantly higher accuracy for the whole population than ASM (Wilcoxon signed-rank test, p<0.001) and similar accuracy to TbM (Wilcoxon signed-rank test, not significant).

### Template

**Fig 3:**
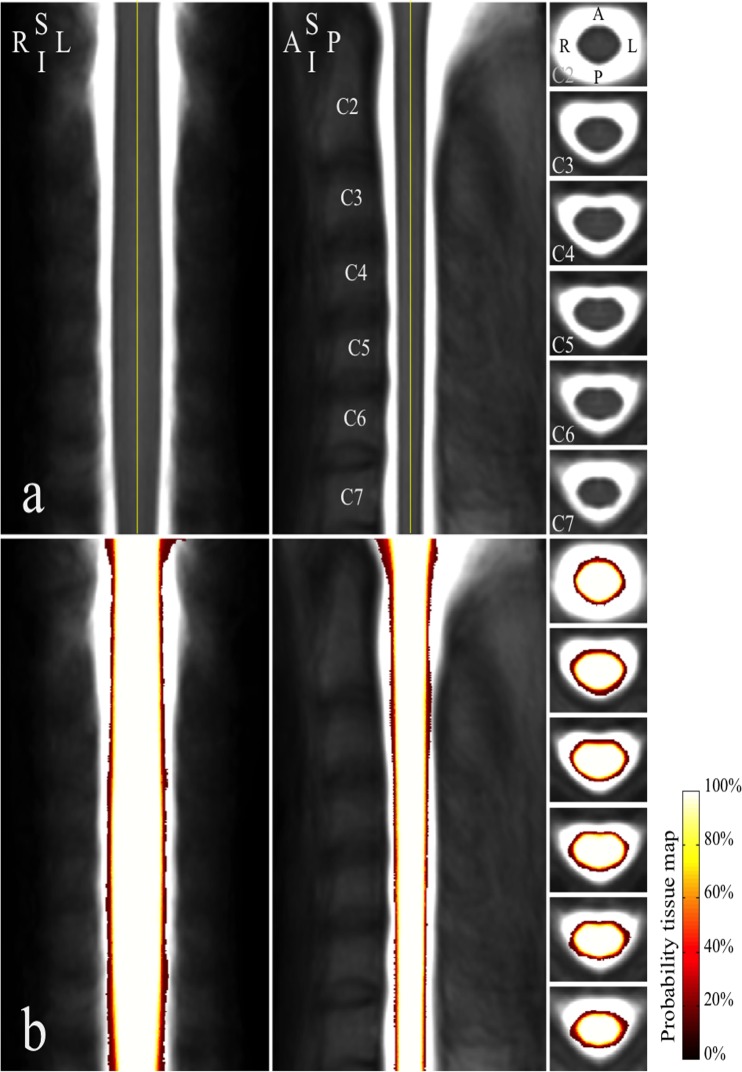
Cervical spinal cord template and probability tissue map. (a) Coronal (left), sagittal (middle) and axial views (right) of the cervical spinal cord template through mid-vertebral level from C2 to C7. (b) Probability tissue map. Views are arranged in the same order and orientation as in (a). A, anterior; I, inferior, L, left, P, posterior, R, right; S, superior.

Fig. 2 shows an example of cord straightening for images from an ALS patient. Visual evaluation revealed an asymptomatic syrinx in the MR images of one healthy subject, who was subsequently excluded from template construction, leading to a final group of 59 healthy volunteers (mean age ± SD: 44.6 ± 16.2 years, range: 21–72 years, 31 females). During the template construction step, the experienced operator had to adjust visually poor segmentations in rare occasions (error rate = 2.57%, defined as: 100 × (the number of mis-segmented axial slices)/(the total number of segmented axial slices)) using TbM [18]. Finally, images from all subjects were centered and straightened. Template and probability tissue map of the cervical spinal cord for the 59 healthy subjects are shown in Fig. 3.

## Discussion

DTbM accuracy was evaluated visually by a neuroradiologist at the cervical and thoracic spinal cord levels and compared with ASM. Besides, DTbM accuracy was visually and quantitatively evaluated at the cervical and thoracic levels and compared with ASM. In addition, DTbM accuracy was quantitatively evaluated at the C2 vertebral level and compared with ASM and the well-established method for CSA measurements, namely TbM. A template and a tissue probability map of the cervical spinal cord were constructed.

### Comparison of segmentation methods

DTbM showed higher accuracy for segmenting both the normal and the pathological spinal cord than ASM for the cervical and thoracic spinal cord levels. The lower accuracy of ASM can be explained by the fact that this method relies on hypotheses that are not fulfilled in the pathological spinal cord. These hypotheses are that (a) the cross-sectional shape of the cord varies only slowly in the superior-inferior direction and (b) in cross-sections, the radius from the estimated center to the edges varies smoothly around the edge [14]. Such hypotheses are valid when considering short regions of the spinal cord, for instance C2/C5 vertebral levels where the method has been validated and used to quantify atrophy in MS patients [14]. However, when considering large regions of the spinal cord (Table 1), the first hypothesis is no more valid even in healthy subjects as shown in post-mortem studies [48, 49] and recently in vivo [50]. In neurodegenerative diseases, ALS is characterized by cell loss in the anterior horn and corticospinal tract degeneration [22]. Corticospinal tract degeneration induces atrophy in the lateral direction and loss of alpha motor neurons results in atrophy in the ventral direction. SMA is characterized by degeneration of cell bodies in the anterior horn [21], which potentially induces a pronounced ventral atrophy. In SCI patients, more pronounced atrophy was reported in the antero-posterior direction than in the left/right direction at C2 vertebral level [11]. Furthermore, trauma induced large spinal cord shape deformation at lesion level and around it. Therefore the second hypothesis may not be valid in ALS, SMA and SCI patients and this could explain the lack of ASM accuracy in segmenting pathological populations. As DTbM depends only on image contrast, it has the advantage of being less sensitive to variations in spinal cord shape as compared with ASM.

Quantitative evaluation of DTbM and ASM compared with manual segmentation was not possible for volumetric approach in this study due to the large number of slices to be segmented. For this reason, we resorted to a visual evaluation strategy. In one control subject, visual evaluation revealed a central and focal T_2_-hypersignal at C5 vertebral level corresponding to an asymptomatic syrinx. This subject was therefore excluded from template construction. Furthermore, accuracy results of the visual evaluation were confirmed by the quantitative evaluation of CSA measurement by using DTbM and ASM at the middle of each available spinal cord vertebral level (Table 1, Table 4). The lower accuracy at the thoracic level than at the cervical level could be explained by the lower signal-to-noise ratio inherent to the thoracic coil compared with the cervical coil as well as the choice made for shimming box positioning to focus on cervical and upper thoracic levels [2, 3, 5, 10].

A quantitative evaluation at C2 vertebral level in healthy and pathological subjects was also achieved (Table 5). Even at C2 vertebral level, our analysis showed a higher accuracy of DTbM compared with ASM and similar accuracy for DTbM and TbM. ASM showed improved accuracy than previously reported in [14]. After data preprocessing, DTbM was fully automatic and hence operator independent, which is not the case for TbM and ASM [14, 18, 30, 51].

In regions where CSF spaces were reduced (i.e. regions of narrow spinal canal), Otsu’s global thresholding method failed to extract the CSF correctly. Thus, global contrast enhancement was needed. However, this strategy did not enable initializing contours optimally with TbM leading to misestimating the spinal cord region [30]. In case this strategy failed, correction step S7 and reduction in nerve roots contribution and in the small T_2_-hyperintense region in the final segmentation result were useful (1 control, 1 ALS, 4 SCI patients). However, segmentation and correction step S7 failed in regions of large areas of T_2_-hyperintensity in the spinal cord (1 SMA patient), large regions of spinal canal narrowing with limited CSF space (1 SMA patient) as well as at the lesion level in 4 SCI patients with T_2_-hypersignal and atrophy of the spinal cord and narrow spinal canal.

### Template construction

DTbM was used to construct a spinal cord template. Despite the high spatial resolution of imaging data, we were not able to determine the precise position of the nerve roots in all subjects, which would ideally enable to standardize all subjects spinal level by spinal level. Alternatively, two ways of standardization can be considered: 1) standardization to the whole median cord length [25]; 2) standardization to the median vertebral level for each vertebral level. As demonstrated previously for the cervical spinal cord [52], nerve root position showed less variation than vertebral body position. Therefore, the first strategy was expected to better preserve nerve roots position than the second. Furthermore, this strategy enabled to remove the effect of cord length in the standardized images [25]. Compared with previous studies that used ASM to construct low-resolution 1.5T MRI cervical spinal cord templates [25, 27, 28], the segmentation method proposed here using high-resolution 3T MR images showed improved accuracy. In addition, the new method resulted in a centered and straight template and probability tissue map.

In a recent study, a spinal cord template from 3T T2-weighted MR images and a small sample of 16 subjects was proposed with minimal manual intervention [26]. The template included the cervical and mid-thoracic regions (i. e., from C2 to T6 vertebral levels). MR images were segmented using a graph-cut segmentation method that is known to lack validation [53]. The standardization procedure consisted of a nonlinear registration of each subject’s spinal canal into an average space as it is usually done when spatially normalizing brain MR images [54–56]. Given the small size of the spinal cord compared with CSF as well as the high inter-subject CSA variability for CSF in comparison to the spinal cord [57], nonlinear registration of the whole spinal canal is expected to increase bias when registering subjects into an average space, while in the present study we focussed on standardizing the spinal cord only. Unlike what we proposed, the standardization procedure proposed in [26] did not take inter-individual nerve roots position variability into account [58]. Besides, the error in CSA area estimation using nonlinear registration (mean error = 16%) would make the method too inaccurate for atrophy quantification in the pathological context [2, 3, 5, 10].

### Atrophy quantification in neurodegenerative diseases and trauma

Grey and white matter could not be differentiated in the acquired images due to insufficient contrast inside the spinal cord. However, as mentioned before, atrophy may predominate in a particular direction in neurodegenerative diseases and trauma. This preferential direction of cord atrophy may be detected in group analysis using surface-based morphometry techniques on spinal cord masks normalized to the template space instead of using voxel-based morphometry. For instance, morphological changes have been successfully detected in the hippocampus and ventricles in Alzheimer’s disease using radial distance approach and tensor-based morphometry [59, 60] and in the lateral ventricles in HIV/AIDS patients using multivariate tensor-based morphometry [61], as well as in the basal ganglia in ALS patients using surface-based vertex approach [62]. Our segmentation method may also measure accurately CSA and spinal cord volumes in a large range of pathologies.

### Limitations and future directions

Manual outlining was taken as the ground truth for methods evaluation, however the accuracy of this procedure strongly depends on the operator’s experience degree [63]. Nevertheless, we believe that segmentations performed by the trained operator (four years’ experience) are close to the ground truth and therefore bias should be minimal. Future studies could use majority voting or simultaneous truth and performance level estimation methods to produce ground truth segmentation for method evaluation [64, 65].

We did not have the opportunity to conduct a scan-rescan evaluation of DTbM. However, DTbM is based on the well-established method TbM [18], which showed low scan-rescan variability [29]. Thus, DTbM is expected to have the same order of variability as TbM, but this is worth further investigating.

Future studies could also integrate two anatomical imaging acquisitions for accurate nerve root localization [66] and for white and grey matter delineation using high-resolution T_2_*-weighted 2D gradient recalled echo sequence, a multi-echo gradient-echo sequence (MGE) or a gradient echo with a multi-shot echo-planar imaging sequence (GE-MS EPI) [67, 57]. For instance, MGE sequence has already enabled construction of a probabilistic cervical and thoracic spinal cord atlas of 15 subjects [57]. However, because of acquisition time constraint, only one slice per vertebral level was acquired (0.5×0.5 mm^2^ in-plane resolution and 5 mm slice thickness). Furthermore, manual segmentation was used for grey and white matter delineation, a procedure that is time consuming and the accuracy of which strongly depends on the operator’s experience degree [30, 63]. GE-MS EPI sequence also enabled the construction of an atlas of the cervical spinal cord. However, multiple manual outlining was needed to construct a “gold standard” segmentation, which is extremely time consuming [68], despite the benefit in accuracy. As T_2_*-weighted images present a spinal cord/CSF contrast similar to that of T_2_-weighted images, DTbM would likely be able to segment the spinal cord accurately using T_2_*-weighted data. Grey matter could be extracted from such images using an atlas-based segmentation approach via the template from Taso et al. (2014). Resulted masks from T2-weighted and T2*-weighted images could be fused to construct a probabilistic spinal cord atlas of white and grey matter as recently proposed in [26].

Besides, as DTbM segments each axial slice independently, parallelization of segmentations could be envisaged [69], which would drastically reduce time computation (few milliseconds per slice).

## Conclusion

We developed a fast and accurate semi-automated segmentation method of the spinal cord at both cervical and thoracic levels from 3T T2-weighted MR images, applied to healthy subjects and patients with various spinal cord diseases. DTbM showed higher accuracy than ASM and similar accuracy to TbM, however it clearly facilitated the whole processing by limiting the manual intervention. This enabled the construction of a cervical spinal cord template and tissue probability map.

## Supporting Information

S1 Fig Illustration of the behaviour of DTbM method. Good agreement of DTbM method: (a,b) in a spinal cord region with T2-hypersignal in a SCI patient; (c,d) in an atrophied spinal cord region in an ALS patient; (e,f) in a narrow spinal canal region in an SMA patient. Failure of DTbM method: (g,h) in a region with T2-hypersignal in a SCI patient; (i,j) in an atrophied spinal cord region in an ALS patient; (k,l) in a narrow spinal canal region in an ALS patient. A, anterior; I, inferior, L, left, P, posterior, R, right; S, superior.
(TIFF)

S1 Table Results of the individual visual scoring. (XLSX)
