# Supplementary figures and images for: Fast and Accurate Semi-Automated Segmentation Method of Spinal Cord MR Images at 3T Applied to the Construction of a Cervical Spinal Cord Template
Source: PLoS One. 2015 Mar 27;10(3):e0122224. doi: 10.1371/journal.pone.0122224 (PMC4376938; doi:10.1371/journal.pone.0122224)

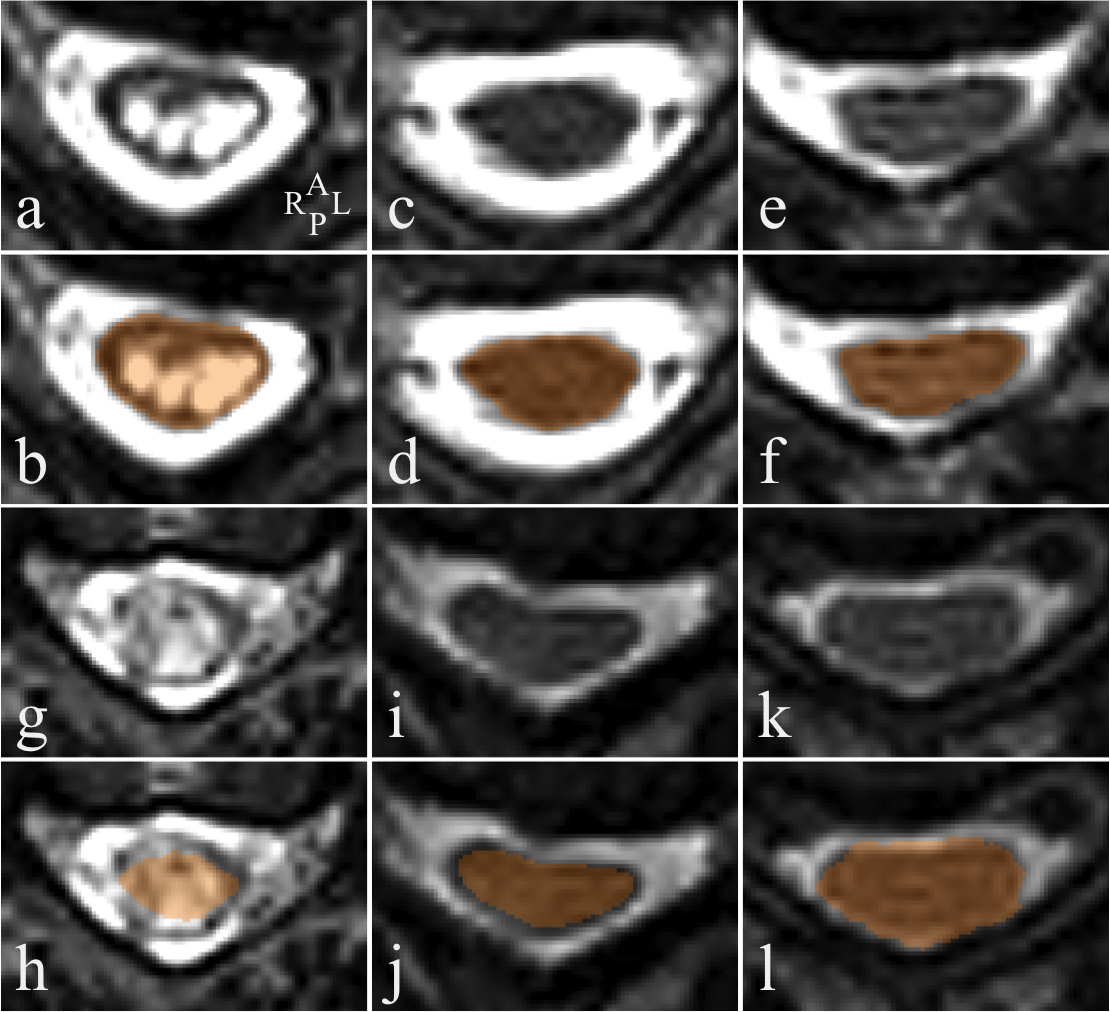

Supplement: S1 Fig — Good agreement of DTbM method: (a,b) in a spinal cord region with T2-hypersignal in a SCI patient; (c,d) in an atrophied spinal cord region in an ALS patient; (e,f) in a narrow spinal canal region in an SMA patient. Failure of DTbM method: (g,h) in a region with T2-hypersignal in a SCI patient; (i,j) in an atrophied spinal cord region in an ALS patient; (k,l) in a narrow spinal canal region in an ALS patient. A, anterior; I, inferior, L, left, P, posterior, R, right; S, superior. (TIFF) [file pone.0122224.s001.tiff]
